# Impact of an educational intervention on dental professionals’ ability to associate oral cancer risk factors with pathological changes: a before-and-after quasi-experimental study

**DOI:** 10.3389/froh.2026.1690796

**Published:** 2026-06-15

**Authors:** Bhoomija Chauhan, Xia Li, Michael McCullough, Nicola Cirillo, Elizabeth Fitriana Sari

**Affiliations:** 1 Dentistry and Oral Health Discipline, Department of Rural Clinical Science, La Trobe Rural Health School, La Trobe University, Bendigo, VIC, Australia; 2 Department of Mathematical and Physical Sciences, La Trobe University, Bundoora, VIC, Australia; 3 Melbourne Dental School, The University of Melbourne, Melbourne, VIC, Australia; 4 College of Dentistry, Ajman University, Ajman, United Arab Emirates

**Keywords:** education intervention, general dental practitioner, oral cancer risk factors, oral cancer screening, oral potentially malignant disorders

## Abstract

**Background:**

Early detection of oral cancer depends on dental professionals’ ability to recognise both risk factors and pathological changes in the oral mucosa. Educational interventions may enhance this diagnostic awareness, particularly in settings where exposure to oral cancer cases and specialised training opportunities may be limited.

**Objective:**

This study aimed to evaluate the impact of an educational intervention on dental professionals’ ability to associate oral cancer risk factors with pathological changes.

**Methods:**

A before-and-after quasi-experimental study design was used. General dental practitioners from multiple regions in Indonesia participated in an oral cancer educational programme. Participants completed questionnaires before and after the intervention assessing their knowledge of oral cancer risk factors and oral potentially malignant disorders. The primary outcome was defined as the change in correlation between correctly identified risk factors and pathological changes. Kendall's tau-*b* correlation coefficients were calculated, and differences between correlations were assessed using Fisher's *r*-to-*z* transformation.

**Results:**

A total of 177 responses were obtained before the intervention and 144 responses after the intervention. The correlation between identified oral cancer risk factors and pathological changes increased slightly from 0.464 [95% confidence interval (CI): 0.383–0.538] before the intervention to 0.485 (95% CI: 0.397–0.565) after the intervention. However, the difference between the correlations was not statistically significant (*p* = 0.8109).

**Conclusion:**

The educational intervention was associated with a modest increase in dental professionals’ ability to associate oral cancer risk factors with pathological changes, although the change in correlation was not statistically significant. These findings suggest that educational initiatives may contribute to improving diagnostic awareness, but more comprehensive or repeated training strategies may be required to produce measurable improvements.

## Introduction

Oral cancer is one of the most common neoplasms of the head and neck, encompassing lesions of the oropharynx, oral cavity, and lip (1). It is a disease of high mortality, with global deaths increasing substantially between 1990 and 2019, as reported in global burden estimates (2). Persistently high mortality is also reflected in Global Cancer Observatory (IARC) data for 2022 (3). In Indonesia, oral cancer mortality rose by 144.85% during this period (2), making it a pressing national health challenge. Importantly, oral cancer is typically preceded by clinically detectable pathological changes, collectively known as oral potentially malignant disorders (OPMDs). These conditions, including leukoplakia, erythroplakia, and oral submucous fibrosis, carry varying risks of malignant transformation depending on lesion type and the influence of specific risk factors. Accurate and timely recognition of OPMDs is therefore essential, as it enables clinicians to determine prognosis, select appropriate management strategies, and intervene early to mitigate progression.

Despite this, evidence shows that most oral cancer cases in Indonesia are diagnosed late. Among hospital admissions, 82.2% present at an advanced stage, underscoring the persistent delay in detection (4). This suggests that dentists in Indonesia lack the ability to associate risk factors with pathological changes. Although new diagnostic techniques and knowledge about risk factors (5) have expanded, there has been no significant improvement in survival rates over the past 5 years (6). The high prevalence of three main risk factors—smoking, betel quid chewing, and alcohol use—in Indonesia (7) further emphasises the need for general dental practitioners to detect important pathological changes like leukoplakia, erythroplakia, and oral submucous fibrosis early and associate them with risk factors (8).

The importance of early detection cannot be overstated. According to the World Health Organization (WHO), timely diagnosis of oral cancer or OPMDs enables minimally invasive surgical interventions, which significantly improve prognosis (9). Early detection also provides an opportunity to raise awareness among patients about modifiable risk factors, encourage preventive behaviours, and promote regular dental visits (7). Conversely, late detection results in extensive surgical procedures with poor outcomes, increases treatment costs, and places a significant financial burden on both the healthcare system and the population. In heavily populated countries such as Indonesia, these factors also translate into broader economic consequences (2).

Several OPMDs, such as proliferative verrucous leukoplakia and erythroplakia, are recognised for their high malignant potential and require vigilant screening (10) and early intervention (11). Nevertheless, the extent to which general dental practitioners possess the necessary knowledge to carry out effective screening remains unclear. For example, a study by Wimardhani et al. explored Indonesian general dental practitioners’ knowledge of oral cancer risk factors and diagnostic ability and found considerable knowledge gaps (7). The study also reported a low response rate of 49.2%, which limits the representativeness of the findings. Importantly, prior research has primarily documented baseline knowledge levels but has not tested interventions designed to enhance general dental practitioners’ ability to associate pathological changes with risk factors. As a result, evidence regarding the effectiveness of educational interventions in improving general dental practitioners’ diagnostic capacity remains absent.

To address this gap, the present study aimed to evaluate whether an educational intervention could improve dental professionals’ ability to associate oral cancer risk factors with pathological changes, representing an essential component of diagnostic awareness for early detection.

This dual focus not only contributes new knowledge to the Indonesian context but also provides insights that are globally relevant, including for countries such as Australia, where literature on oral cancer screening education remains limited. By identifying gaps in knowledge and testing interventions to address them, this study may also inform improvements to undergraduate dental curricula and continuing professional development (CPD) programmes. Ultimately, such initiatives could strengthen the capacity of general dental practitioners to detect oral cancer earlier, reduce the burden of late-stage disease, and improve oral health outcomes in a systematic manner.

## Methodology

### Study design

The data collection and main results of the original cross-sectional study were previously published by Sari et al. (12). The present study extends that work by employing a before-and-after quasi-experimental design to evaluate the impact of a structured educational intervention on the ability of dental professionals to associate oral cancer risk factors with pathological changes.

**Figure 1:**
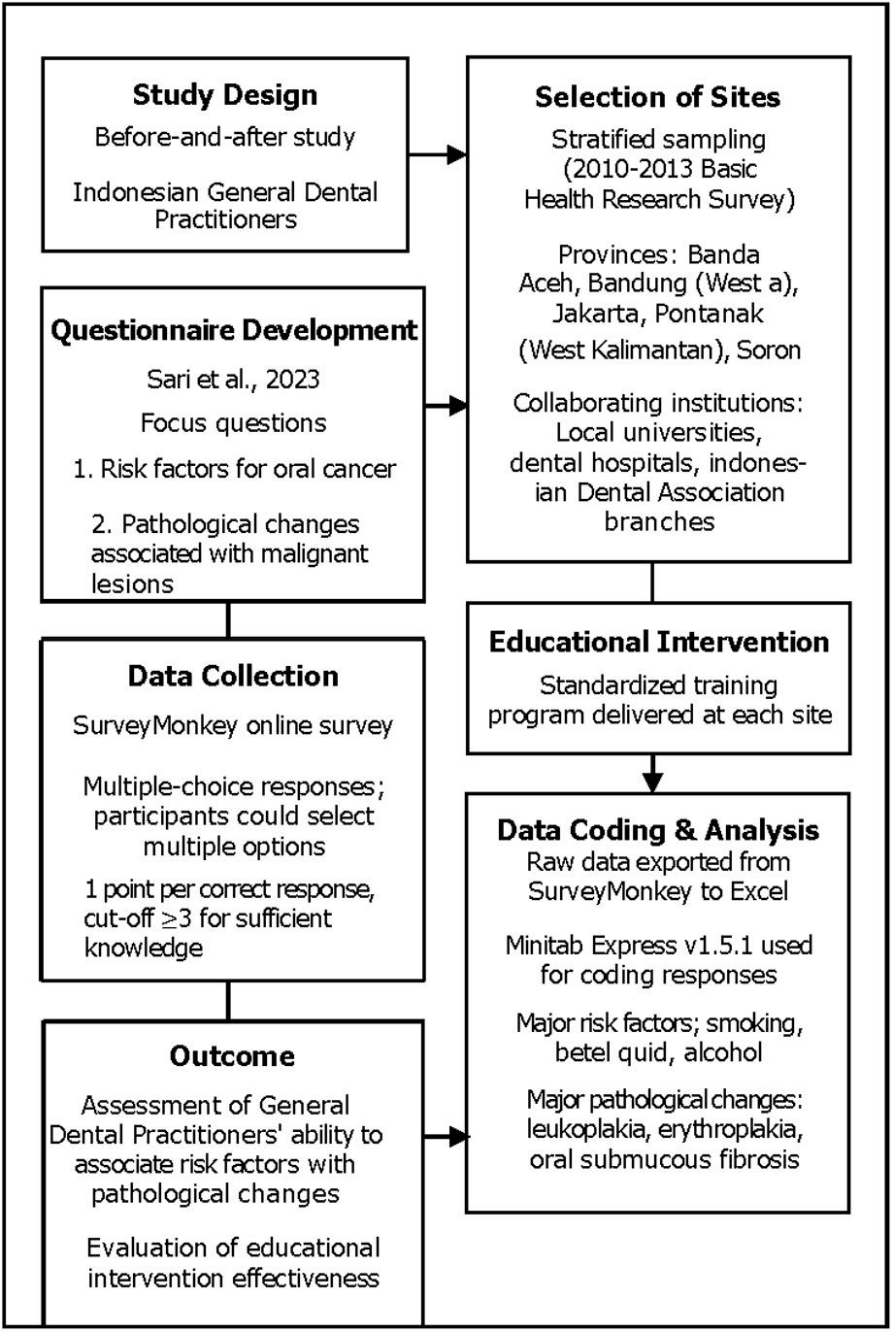
Visual diagram of study methodology.

Baseline data were collected prior to the educational programme, and follow-up data were obtained immediately after completion of the intervention using the same questionnaire developed by Sari et al. (12). The primary outcome was defined as the change in the correlation between correctly identified oral cancer risk factors and correctly identified OPMDs. This design allowed assessment of baseline knowledge and the impact of the intervention. The study design is illustrated in Figure 1.

### Participants and study setting

Participants were general dental practitioners practicing in Indonesia who attended an oral cancer educational programme conducted across five provinces: Banda Aceh, Bandung (West Java), Jakarta, Pontianak (West Kalimantan), and Sorong (West Papua). Provinces were selected through stratified sampling based on the 2010–2013 Basic Health Research Survey, considering risk factor prevalence, sex ratio, socioeconomic status, and urbanisation. The training sessions were organised in collaboration with local universities, dental hospitals, and regional branches of the Indonesian Dental Association.

Participants represented a range of professional settings, including university-based clinicians, hospital-based dentists, and private practitioners. Participation in the study was voluntary, and respondents completed the questionnaires anonymously. A total of 177 responses were collected before the intervention, and 144 responses were obtained after the intervention, representing an attrition rate of 18.6% between the two survey rounds.

### Questionnaire

Data were collected using a structured questionnaire designed to assess participants’ knowledge of oral cancer risk factors and OPMDs. The study utilised a validated survey developed by Sari et al. (12). Two focus questions were analysed:

1. Recognition of oral cancer risk factors: Participants were asked to identify major behavioural and environmental risk factors associated with oral cancer, including smoking, betel quid chewing, and alcohol consumption.
2. Recognition of pathological changes/OPMDs: Participants were asked to identify pathological changes associated with oral cancer development, including leukoplakia, erythroplakia, and oral submucous fibrosis.

Participants were required to associate these risk factors with relevant pathological conditions. The questionnaire was administered both before and after the educational intervention to evaluate changes in participants’ understanding.

Responses were multiple-choice, with one point awarded per correct response. A score of ≥3 correct answers indicated sufficient knowledge. This threshold reflected recognition of the three major oral cancer risk factors prevalent in Indonesia (smoking, betel quid use, and alcohol consumption) as well as the three key OPMDs relevant to early detection (leukoplakia, erythroplakia, and oral submucous fibrosis). This threshold was consistent with prior studies conducted by van der Waal in 2009 (11) and Wimardhani in 2021 (7), which stated the most prevalent risk factors and pathological changes in Indonesia. The reliability and validity of the questionnaire were confirmed in a pilot study conducted with 10 Indonesian general dental practitioners.

### Educational intervention

The educational intervention consisted of a structured oral cancer training programme delivered to general dental practitioners.

The educational programme consisted of two main components:

1. Theoretical component: This component included lectures and interactive discussions covering the epidemiology of oral cancer, major behavioural risk factors prevalent in Indonesia (including tobacco use, betel quid chewing, and alcohol consumption), and clinical features of OPMDs.
2. Clinical awareness component: This component incorporated case-based learning using clinical images and representative case scenarios of OPMDs. Participants were guided in the identification of lesion characteristics, differentiation of pathological changes, and interpretation of clinical findings. These sessions emphasised the association between observable mucosal changes and underlying risk factors, thereby supporting the development of diagnostic reasoning.

Following these sessions conducted in clinical settings, participants underwent practical training in performing conventional oral examinations (COEs). General dental practitioners were calibrated on standardised examination techniques to ensure consistency in clinical assessment.

In the subsequent phase of training, participants applied these skills in real clinical settings under the supervision of the principal investigator and local oral medicine specialists. They performed early detection of oral cancer through COEs on patients attending primary healthcare facilities for 1 day. This hands-on component was designed to reinforce learning and enhance participants’ clinical competence in oral cancer screening.

### Data analysis

Survey responses were collected via SurveyMonkey, coded in Excel, and analysed using Minitab Express v1.5.1. Scoring for risk factors and pathological changes ranged from 0 (none identified) to 4+ (all major and additional factors identified).

Data were analysed using statistical methods appropriate for ordinal and categorical variables. The data were distributed using two contingency tables to illustrate the relationship between responses on risk factors and pathological changes before and after the educational intervention (independent variable). The association between recognition of oral cancer risk factors and pathological changes was assessed using Kendall's tau-*b* correlation coefficient. This statistical measure was chosen because it evaluates the strength and direction of association between two ordinal variables (13).

To evaluate the impact of the educational intervention, correlations obtained before and after the intervention were compared using Fisher's *r*-to-*z* transformation. The proposed level of statistical significance was *p* < 0.05, rejecting the null hypothesis that the educational intervention on oral cancer screening had no significant impact on the association between risk factors and pathological changes in Indonesian general dental practitioners (14).

Correlation strength was interpreted using commonly accepted thresholds in biomedical research, with values between 0.40 and 0.60 indicating a moderate association.

Because the post-intervention survey included fewer responses than the pre-intervention survey, analyses were conducted using a per-protocol approach based on available responses at each time point. Correlation analysis was used to evaluate the extent to which participants were able to associate oral cancer risk factors with pathological changes, reflecting an integrated understanding of these two domains rather than independent recognition of each variable.

### Ethics

Ethics approval was granted by the Faculty of Medicine, University of Padjadjaran, Health Research Ethics Committee, Indonesia (reference number 701/UN6.C1.3/KEPK/PN/2016), and by The University of Melbourne (Ethics reference ID: 1748812).

## Results

A total of 177 Indonesian general dental practitioners participated in the standardised training programme, which was conducted across five regions in Indonesia: Banda Aceh, West Papua, Jakarta, West Kalimantan, and West Java. The majority of participants were from West Java, comprising 35.59% before the intervention and increasing to 38.19% afterwards. Notably, 88.7% of participants were female, while 11.3% were male prior to the intervention.

Baseline data collected before the educational intervention are presented in a contingency table (Table 1), which illustrates the correlation between the identification of risk factors (categorised as fewer than three, three, and more than three) and the detection of OPMDs (similarly categorised). The analysis included a total of 177 responses.

**Table 1:** Association between indicated risk factors and OPMDs before the educational intervention.

		Indicate OPMD (number and % of correct responses)		
		Less than 3	Equal to 3	More than 3
Indicate risk factor (number and % of correct responses)	Less than 3	55 (31.07%)	3 (1.69%)	1 (0.56%)
	Equal to 3	42 (23.73%)	13 (7.34%)	2 (1.13%)
	More than 3	26 (14.69%)	3 (1.69%)	32 (18.08%)

Prior to the educational intervention, the ability of general dental practitioners to correlate risk factors with pathological changes was limited. As illustrated in Table 1, most responses (*n* = 55, 31.07%) indicated that general dental practitioners were able to associate fewer than three identified risk factors with fewer than three identified OPMDs.

Similarly, the data collected after the educational intervention are presented in Table 2, which illustrates the association between indicated risk factors and OPMD lesions by number of responses. The post-intervention survey consisted of 144 responses. A shift was observed in the distribution of responses shown in Table 2. The majority of respondents demonstrated the ability to associate three or more risk factors with three or more OPMDs (*n* = 83, 57.64%).

**Table 2:** Association between indicated risk factors and OPMDs after educational intervention.

		Indicate OPMD (number and % of correct responses)		
		Less than 3	Equal to 3	More than 3
Indicate risk factor (number and % of correct responses)	Less than 3	10 (6.94%)	2 (1.39%)	3 (2.08%)
	Equal to 3	8 (5.56%)	8 (5.56%)	6 (4.17%)
	More than 3	10 (6.94%)	14 (9.72%)	83 (57.64%)

To assess the correlation between indicated risk factors and OPMDs before the educational intervention, Kendall's tau-*b* correlation was used. Table 3 shows a correlation coefficient of 0.464 with a significance level of *p* < 0.001, suggesting a strong, meaningful, and positive association between indicated risk factors and OPMDs before the educational intervention.

**Table 3:** Correlation coefficient between indicated risk factors and OPMDs before educational intervention.

Kendall's tau_*b* correlation value			95% confidence interval
		Indicate OPMD (3 levels)	0.383–0.538
Indicate risk factor (three levels)	Correlation coefficient	0.464	
	*p*-value	<0.001	
	Number	177	

Correlation is significant at *p* < 0.001 (two-tailed). Estimation is based on Fisher's *r*-to-*z* transformation.

Similarly, to assess the correlation after the educational intervention, Kendall's tau-*b* was calculated. Table 4 shows a correlation coefficient of 0.485 with a significance level of *p* < 0.001, suggesting a strong, meaningful, and positive correlation between identified risk factors and OPMDs after the educational intervention.

**Table 4:** Correlation coefficient between indicated risk factors and OPMDs after educational intervention.

Kendall's tau_*b* correlation value			95% confidence interval
		Indicate OPMD (3 levels)	0.397–0.565
Indicate risk factor (three levels)	Correlation coefficient	0.485	
	*p*-value	<0.001	
	Number	144	

Correlation is significant at *p* < 0.001 (two-tailed). Estimation is based on Fisher's *r*-to-*z* transformation.

### Comparison of correlations before and after educational intervention

To statistically compare correlations before and after the educational intervention, Fisher-*z* transform was used to calculate the confidence interval and assess significance with a *p* < 0.05 (14). Comparison of the two correlations as independent groups showed the there was no significant difference between the two Kendall's tau-*b* values, with *z* = −0.2392, *p*-value = 0.8109. Therefore, findings indicate that there was no significant increase in correlation following the intervention.

## Discussion

This study evaluated the impact of an educational intervention on dental professionals’ ability to associate oral cancer risk factors with pathological changes. The results demonstrated a modest increase in correlation between these two domains. However, the difference between the pre- and post-intervention correlations was not statistically significant. These findings suggest that while the intervention may have improved participants’ awareness of oral cancer-related factors, the change in the primary outcome should be interpreted with caution.

Educational interventions often lead to immediate improvements in knowledge following training programmes (12). However, knowledge recall alone does not necessarily translate into improved diagnostic reasoning. In the present study, the primary outcome focused on the association between recognition of oral cancer risk factors and identification of pathological changes, as this relationship reflects a deeper level of clinical understanding required for the early detection of oral cancer.

Our findings demonstrate that following the educational intervention, general dental practitioners’ ability to link risk factors with pathological alterations improved, as indicated by the increase in Kendall's tau-*b* value from 0.464 to 0.485. A cut-off score for general dental practitioners’ performance was defined as the detection of at least three risk factors and three pathological alterations, as presented in Tables 1, 2. For the purpose of analysis, a cut-off score was defined as the identification of at least three oral cancer risk factors and three pathological alterations. This threshold was selected because it represents recognition of the three major oral cancer risk factors in Indonesia (alcohol consumption, smoking, and betel quid use) and the three key oral potentially malignant disorders (oral submucous fibrosis, erythroplakia, and leukoplakia), which are critical for early detection and intervention (7, 8).

Prior to the educational intervention, participants’ ability to associate identified risk factors with pathological changes related to OPMDs was poor. The majority of participants (31.07%) linked fewer than three risk factors to fewer than three pathological changes (OPMDs), as shown in Table 1.

The observed pattern may reflect several contextual factors. First, dental practitioners may have limited exposure to oral cancer cases in routine clinical practice, which could affect their ability to associate specific risk factors with corresponding pathological changes. Second, oral cancer education within undergraduate dental curricula may vary across institutions, potentially resulting in differences in baseline knowledge among practitioners. Third, continuing professional education opportunities specifically focused on oral cancer detection and risk assessment may be limited in certain regions.

There was a strong association between the identification of risk variables and pathological alterations (Kendall's tau-*b* = 0.464, *p* < 0.001), as seen in Table 3. This implies that participants’ lack of knowledge was consistent across both domains (OPMDs and indicated risk factors), highlighting the substantial connection between theoretical understanding of risk factors and clinical oral manifestations, which general dental practitioners should be made aware of through educational interventions.

After the educational intervention, 57.64% of participants were able to associate more than three pathological abnormalities (OPMDs) with more than three identified risk factors (Table 2). The findings presented in Table 4 further support the relationship between the two domains, showing a significant positive correlation between the identification of oral cancer risk factors and pathological changes after the educational intervention (Kendall's tau-*b* = 0.485, *p* < 0.001).

Although there was a slight increase from 0.464 to 0.485 in the correlation coefficient describing the association between risk factors and pathological changes (OPMDs) following the educational intervention, this difference was not statistically significant (*z* = −0.2392, *p* = 0.8109) based on Fisher's *z* transformation (14).

Despite the lack of statistical significance, the observed increase may still hold practical value. It represents an important step towards strengthening future educational interventions aimed at reinforcing knowledge and improving confidence among general dental practitioners in the timely identification of OPMDs. As shown in Table 2, there was a notable improvement in the ability of general dental practitioners to associate risk factors with pathological changes, with more than half of the participants exceeding the defined threshold. This finding suggests that educational initiatives may still play an important role in strengthening clinicians’ understanding of oral cancer risk factors and related pathological changes, particularly in settings where structured training opportunities are limited.

The study also highlights the importance of integrating both risk factor awareness and recognition of OPMDs within oral cancer education programmes. Effective early detection strategies require dental professionals not only to recognise suspicious lesions but also to understand the behavioural and environmental factors associated with malignant transformation (4, 15, 16).

Similar educational interventions have been implemented in countries such as Germany (17) and India (16), where participants demonstrated statistically significant improvements in their ability to screen for oral cancer (4, 15, 16). The present study therefore provides an important baseline for educational interventions aimed at improving oral cancer detection in Indonesia, where published evidence is limited.

### Limitations and strengths

This study had several limitations. The limited sample size does n't allow generalisation of the findings, thus reducing the power and contributing to the lack of statistically significant results (18). The number of responses decreased from 177 in the pre-intervention survey to 144 in the post-intervention survey, representing 18.6% attrition. This reduction in sample size may introduce attrition bias, particularly if individuals who did not complete the post-intervention questionnaire differed systematically from those who did. Consequently, the analyses were conducted using a per-protocol approach based on available responses at each time point.

Despite the lack of statistical significance, the observed increase in correlation following the educational intervention may still have practical importance. The upward trend suggests that the intervention may have contributed to improving general dental practitioners' understanding of oral cancer risk factors and oral potentially malignant disorders (OPMDs), thereby supporting their ability to identify and screen for these conditions more effectively. This indicates that educational intervention does help increase the knowledge of general dental practitioners and can help them screen oral cancer and OPMDs better. The improvement in general dental practitioners’ understanding provides us with a framework for tailoring education to strengthen their knowledge of oral cancer, OPMDs, and risk factors.

This study further highlights the critical role of continuing professional education in strengthening early detection of oral cancer and OPMDs. Evaluating the effectiveness of educational interventions is essential to ensure that training programmes are not only informative in terms of theoretical knowledge acquisition but also clinically meaningful and capable of improving diagnostic reasoning and decision-making in real practice settings.

Importantly, educational intervention studies such as this provide evidence to guide the development and implementation of more effective educational strategies, helping institutions optimise training delivery in terms of both time efficiency and financial investment. By identifying which educational components contribute most effectively to improvements in knowledge, confidence, and clinical performance, this research may assist in designing future interventions that are more targeted, practical, scalable, and cost-effective.

### Implications for future research and relevance to Australia

This study paves the way for future research with larger sample sizes to better evaluate the effect of educational interventions the general dental practitioners. Moreover, longitudinal trials can be conducted over longer periods of time to assess the long-term impacts of educational intervention.

While the present study focussed on Indonesian general dental practitioners, its findings are globally relevant in other countries with significant oral cancer burdens. For example, in Australia, oral cancer remains an important public health concern. According to Oral Health Services, Victoria (OHV) (19), approximately 16 new diagnoses of oral cancer are made on average every week in Victoria alone. Those who use tobacco and consume alcohol are at a significantly greater risk. Other risk factors include prolonged UV radiation exposure and Indigenous Australian status.

However, a study showed that Australian oral health practitioners lack the confidence and training in oral cancer screening (20). Therefore, to reduce the burden on the Australian healthcare system, it is important that general dental practitioners are equipped with current practices and knowledge necessary for timely diagnosis. The findings of this study may inform the development of similar educational interventions in Australia, tailored to the major oral cancer risk factors and oral potentially malignant disorders relevant to the local population. Such interventions could be used to evaluate and strengthen dental practitioners' ability to recognise and appropriately associate risk factors with pathological changes during oral cancer screening.

## Conclusion

The educational intervention demonstrated a modest improvement in dental professionals’ ability to associate oral cancer risk factors with pathological changes. Although the change in the primary outcome did not reach statistical significance, the findings suggest the potential value of targeted educational strategies in strengthening clinicians’ diagnostic awareness.

Importantly, this study provides a practical framework for educational interventions that integrate both theoretical knowledge and hands-on clinical components. Such approaches may support the development of more effective training models for oral cancer risk assessment and early detection. The study also highlights the opportunity for future longitudinal investigations in Indonesia and comparable settings, including Australia.

Further research with larger sample sizes, improved participant retention, and longer follow-up periods is warranted to better evaluate the sustained impact of educational interventions on clinical practice and patient outcomes.

## Data Availability

The original contributions presented in the study are included in the article/Supplementary Material; further inquiries can be directed to the corresponding author.
